# Benzene-1,4-diol–5-(1*H*-imidazol-1-yl)pyrimidine (1/1)

**DOI:** 10.1107/S1600536811043819

**Published:** 2011-10-29

**Authors:** Yan-Ke Jiang, Gui-Ge Hou

**Affiliations:** a Research Center of Medical Chemisty and Chemical Biology, Chongqing Technology and Business University, Chongqing 400067, People’s Republic of China; b College of Pharmacy, Binzhou Medical University, Yantai 264003, People’s Republic of China

## Abstract

The asymmetric unit of title compound, C_7_H_6_N_4_·C_6_H_6_O_2_, contains one 5-(1*H*-imidazol-1-yl)pyrimidine mol­ecule and two half benzene-1,4-diol mol­ecules; the benzene-1,4-diol mol­ecules are located on individual inversion centers. In the pyrimidine mol­ecule, the imidazole ring is twisted with respect to the pyrimidine ring at a dihedral angle of 25.73 (7)°. In the crystal, O—H⋯N hydrogen bonds link the mol­ecules to form supra­molecular chains. π–π stacking is also observed in the crystal, the centroid–centroid distance between parallel imdazole rings being 3.5543 (16) Å.

## Related literature

For related structures, see: Nieuwenhuyzen *et al.* (1999 ▶); Clausen *et al.* (2010 ▶).
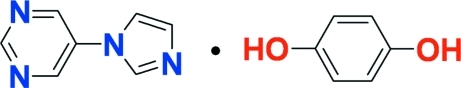


## Experimental

### 

#### Crystal data

C_7_H_6_N_4_·C_6_H_6_O_2_
*M* *_r_* = 256.27
Triclinic, 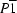

*a* = 6.8219 (18) Å
*b* = 9.550 (3) Å
*c* = 10.449 (3) Å
α = 108.177 (3)°
β = 102.381 (4)°
γ = 98.602 (4)°
*V* = 614.3 (3) Å^3^
*Z* = 2
Mo *K*α radiation
μ = 0.10 mm^−1^
*T* = 298 K
0.36 × 0.24 × 0.12 mm

#### Data collection

Bruker SMART 1000 diffractometer
3103 measured reflections
2176 independent reflections
1791 reflections with *I* > 2σ(*I*)
*R*_int_ = 0.013

#### Refinement

*R*[*F* ^2^ > 2σ(*F* ^2^)] = 0.045
*wR*(*F* ^2^) = 0.115
*S* = 1.04
2176 reflections
174 parameters
H-atom parameters constrained
Δρ_max_ = 0.13 e Å^−3^
Δρ_min_ = −0.30 e Å^−3^

### 

Data collection: *SMART* (Bruker, 2007 ▶); cell refinement: *SAINT* (Bruker, 2007 ▶); data reduction: *SAINT*; program(s) used to solve structure: *SHELXTL* (Sheldrick, 2008 ▶); program(s) used to refine structure: *SHELXTL*; molecular graphics: *SHELXTL*; software used to prepare material for publication: *SHELXTL*.

## Supplementary Material

Crystal structure: contains datablock(s) global, I. DOI: 10.1107/S1600536811043819/xu5356sup1.cif

Structure factors: contains datablock(s) I. DOI: 10.1107/S1600536811043819/xu5356Isup2.hkl

Supplementary material file. DOI: 10.1107/S1600536811043819/xu5356Isup3.cml

Additional supplementary materials:  crystallographic information; 3D view; checkCIF report

## Figures and Tables

**Table 1:** Hydrogen-bond geometry (Å, °)

*D*—H⋯*A*	*D*—H	H⋯*A*	*D*⋯*A*	*D*—H⋯*A*
O1—H1*A*⋯N1^i^	0.82	1.96	2.764 (2)	168
O2—H2*A*⋯N4^ii^	0.82	2.02	2.835 (2)	174

Symmetry codes: (i) 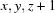; (ii) 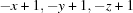.

**Fig. 1.**
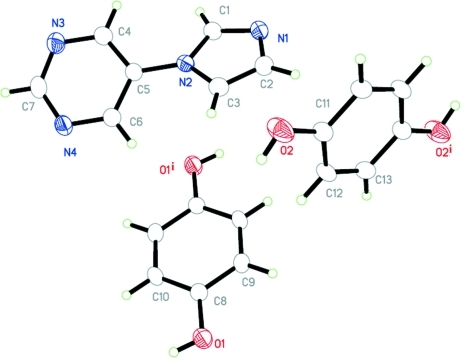
The structure of the title compound with 30% probability displacement ellipsoids.(Symmetry codes: (i) -x,-y + 1,-z + 1)

**Fig. 2.**
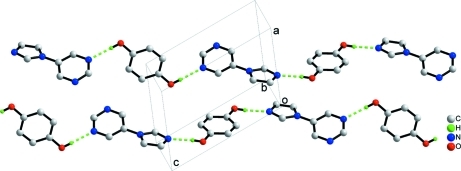
A view of the hydrogen-bonded double-chain observed in the crystal structure of (1).

## supplementary crystallographic information

### Comment

The N atoms on rigid rings, such as pyridine, pyrimidine, imidazole *et al.*, could form strong hydrogen-bond interaction and play an essential role in synthesis of supermolecular compounds. 5-(1*H*-Imidazol-1-yl)pyrimidine (L1) includes three such nitrogen atoms which behave as hydrogen-bond acceptors. benzene-1,4-diol (L2) is a good hydrogen-bonding donor which can form co-crystals with heterocyclic amine systems (Nieuwenhuyzen *et al.*, 1999; Clausen *et al.*, 2010). Here we report the co-crystal states of L1 and L2.

The molecular structure is shown in Fig. 1. The asymmetric unit contains one L1 molecule and two half of L2 in the asymmetric unit. A H-bonding driven double chain was generated from O—H···N hydrogen bonds between these molecules (Fig. 2). Imidazol ring is twisted to pyrimidine ring (the dihedral angle, 25.73 (7)°), while nearly coplanar with benzene ring of L2 (the dihedral angle, 5.54 (7)°). The π–π stacking is also observed in the crystal structure, centroids distance between parallel imdazole ring being 3.5543 (16) Å.

### Experimental

A CH_2_Cl_2_ and CH_3_CN solution (15 ml, 1:1, *v*/*v*) of 5-(1H-imidazol-1-yl)pyrimidine (15.7 mg, 0.1 mmol) and benzene-1,4-diol (11.0 mg, 0.1 mmol) was kept at room temperature. Upon slow evaporation of the solvent about 5 days, colorless crystals were obtained.

### Refinement

All H atoms were placed in idealized positions and treated as riding, with C—H = 0.93 and O—H = 0.82 Å, *U*_iso_(H) = 1.2*U*_eq_(C), or 1.5*U*_eq_(O).

### Crystal data

**Table:**

C_7_H_6_N_4_·C_6_H_6_O_2_	*Z* = 2
*M**_r_* = 256.27	*F*(000) = 268
Triclinic, *P*1	*D*_x_ = 1.385 Mg m^−^^3^
Hall symbol: -P 1	Mo *K*α radiation, λ = 0.71073 Å
*a* = 6.8219 (18) Å	Cell parameters from 1283 reflections
*b* = 9.550 (3) Å	θ = 2.3–26.8°
*c* = 10.449 (3) Å	µ = 0.10 mm^−^^1^
α = 108.177 (3)°	*T* = 298 K
β = 102.381 (4)°	Block, colourless
γ = 98.602 (4)°	0.36 × 0.24 × 0.12 mm
*V* = 614.3 (3) Å^3^	

### Data collection

**Table:**

Bruker SMART 1000 diffractometer	1791 reflections with *I* > 2σ(*I*)
Radiation source: fine-focus sealed tube	*R*_int_ = 0.013
graphite	θ_max_ = 25.2°, θ_min_ = 2.1°
φ and ω scans	*h* = −6→8
3103 measured reflections	*k* = −11→11
2176 independent reflections	*l* = −11→12

### Refinement

**Table:**

Refinement on *F*^2^	Primary atom site location: structure-invariant direct methods
Least-squares matrix: full	Secondary atom site location: difference Fourier map
*R*[*F*^2^ > 2σ(*F*^2^)] = 0.045	Hydrogen site location: inferred from neighbouring sites
*wR*(*F*^2^) = 0.115	H-atom parameters constrained
*S* = 1.04	*w* = 1/[σ^2^(*F*_o_^2^) + (0.0603*P*)^2^ + 0.0979*P*] where *P* = (*F*_o_^2^ + 2*F*_c_^2^)/3
2176 reflections	(Δ/σ)_max_ = 0.002
174 parameters	Δρ_max_ = 0.13 e Å^−^^3^
0 restraints	Δρ_min_ = −0.30 e Å^−^^3^

### Special details

**Table:**

Geometry. All e.s.d.'s (except the e.s.d. in the dihedral angle between two l.s. planes) are estimated using the full covariance matrix. The cell e.s.d.'s are taken into account individually in the estimation of e.s.d.'s in distances, angles and torsion angles; correlations between e.s.d.'s in cell parameters are only used when they are defined by crystal symmetry. An approximate (isotropic) treatment of cell e.s.d.'s is used for estimating e.s.d.'s involving l.s. planes.
Refinement. Refinement of *F*^2^ against ALL reflections. The weighted *R*-factor *wR* and goodness of fit *S* are based on *F*^2^, conventional *R*-factors *R* are based on *F*, with *F* set to zero for negative *F*^2^. The threshold expression of *F*^2^ > σ(*F*^2^) is used only for calculating *R*-factors(gt) *etc*. and is not relevant to the choice of reflections for refinement. *R*-factors based on *F*^2^ are statistically about twice as large as those based on *F*, and *R*- factors based on ALL data will be even larger.

### Fractional atomic coordinates and isotropic or equivalent isotropic displacement parameters (Å^2^)

**Table:**

	*x*	*y*	*z*	*U*_iso_*/*U*_eq_	
C1	0.3093 (3)	0.5951 (2)	0.03876 (18)	0.0443 (4)	
H1	0.3309	0.6809	0.0146	0.053*	
C2	0.2073 (3)	0.3702 (2)	0.02839 (19)	0.0489 (5)	
H2	0.1427	0.2677	−0.0065	0.059*	
C3	0.3175 (3)	0.4481 (2)	0.16172 (19)	0.0479 (5)	
H3	0.3432	0.4109	0.2345	0.058*	
C4	0.6323 (3)	0.83862 (19)	0.27343 (19)	0.0468 (5)	
H4	0.6347	0.8351	0.1838	0.056*	
C5	0.5094 (2)	0.71929 (18)	0.28759 (17)	0.0379 (4)	
C6	0.5138 (3)	0.7279 (2)	0.42203 (18)	0.0470 (5)	
H6	0.4334	0.6490	0.4355	0.056*	
C7	0.7401 (3)	0.9551 (2)	0.5069 (2)	0.0511 (5)	
H7	0.8208	1.0382	0.5843	0.061*	
C8	−0.0026 (3)	0.41667 (18)	0.58820 (17)	0.0396 (4)	
C9	−0.0960 (3)	0.34868 (19)	0.44598 (18)	0.0465 (5)	
H9	−0.1616	0.2463	0.4087	0.056*	
C10	0.0931 (3)	0.56915 (19)	0.64137 (18)	0.0459 (5)	
H10	0.1561	0.6168	0.7371	0.055*	
C11	0.1916 (3)	0.0311 (2)	0.09527 (17)	0.0416 (4)	
C12	0.0237 (3)	0.06699 (19)	0.14148 (17)	0.0425 (4)	
H12	0.0393	0.1122	0.2370	0.051*	
C13	−0.1671 (3)	0.03627 (19)	0.04686 (17)	0.0415 (4)	
H13	−0.2791	0.0609	0.0788	0.050*	
N1	0.2027 (2)	0.46188 (16)	−0.04935 (15)	0.0475 (4)	
N2	0.3850 (2)	0.59439 (15)	0.16903 (14)	0.0397 (4)	
N3	0.7474 (2)	0.95816 (17)	0.38243 (17)	0.0525 (4)	
N4	0.6296 (2)	0.84580 (19)	0.53321 (15)	0.0515 (4)	
O1	−0.0130 (2)	0.33007 (14)	0.67005 (13)	0.0547 (4)	
H1A	0.0532	0.3808	0.7513	0.082*	
O2	0.3846 (2)	0.0619 (2)	0.18407 (13)	0.0668 (4)	
H2A	0.3794	0.0945	0.2655	0.100*	

### Atomic displacement parameters (Å^2^)

**Table:**

	*U*^11^	*U*^22^	*U*^33^	*U*^12^	*U*^13^	*U*^23^
C1	0.0524 (11)	0.0423 (10)	0.0381 (9)	0.0075 (8)	0.0114 (8)	0.0164 (8)
C2	0.0564 (12)	0.0382 (9)	0.0478 (11)	0.0042 (8)	0.0165 (9)	0.0111 (8)
C3	0.0618 (12)	0.0412 (10)	0.0434 (10)	0.0076 (9)	0.0165 (9)	0.0194 (8)
C4	0.0538 (11)	0.0427 (10)	0.0423 (10)	0.0081 (8)	0.0140 (8)	0.0139 (8)
C5	0.0377 (9)	0.0386 (9)	0.0359 (9)	0.0106 (7)	0.0098 (7)	0.0106 (7)
C6	0.0410 (10)	0.0555 (11)	0.0398 (10)	0.0046 (8)	0.0089 (8)	0.0153 (9)
C7	0.0441 (11)	0.0489 (11)	0.0451 (11)	0.0067 (9)	0.0029 (8)	0.0045 (9)
C8	0.0418 (10)	0.0383 (9)	0.0368 (9)	0.0080 (7)	0.0118 (7)	0.0110 (8)
C9	0.0550 (11)	0.0324 (8)	0.0407 (10)	−0.0002 (8)	0.0108 (8)	0.0041 (8)
C10	0.0535 (11)	0.0426 (10)	0.0297 (9)	0.0019 (8)	0.0066 (8)	0.0046 (8)
C11	0.0441 (10)	0.0465 (10)	0.0340 (9)	0.0105 (8)	0.0081 (7)	0.0157 (8)
C12	0.0505 (11)	0.0475 (10)	0.0289 (8)	0.0139 (8)	0.0129 (8)	0.0107 (8)
C13	0.0450 (10)	0.0449 (10)	0.0403 (9)	0.0151 (8)	0.0183 (8)	0.0164 (8)
N1	0.0521 (9)	0.0459 (9)	0.0380 (8)	0.0047 (7)	0.0097 (7)	0.0112 (7)
N2	0.0446 (8)	0.0395 (8)	0.0342 (8)	0.0069 (6)	0.0121 (6)	0.0126 (6)
N3	0.0547 (10)	0.0431 (9)	0.0503 (10)	0.0038 (7)	0.0100 (8)	0.0104 (7)
N4	0.0439 (9)	0.0629 (10)	0.0371 (8)	0.0066 (8)	0.0054 (7)	0.0101 (8)
O1	0.0723 (10)	0.0434 (7)	0.0401 (7)	0.0011 (6)	0.0070 (7)	0.0149 (6)
O2	0.0464 (8)	0.1082 (12)	0.0402 (8)	0.0235 (8)	0.0075 (6)	0.0196 (8)

### Geometric parameters (Å, °)

**Table:**

C1—N1	1.304 (2)	C7—H7	0.9300
C1—N2	1.351 (2)	C8—O1	1.368 (2)
C1—H1	0.9300	C8—C9	1.380 (2)
C2—C3	1.340 (3)	C8—C10	1.382 (2)
C2—N1	1.367 (2)	C9—C10^i^	1.378 (2)
C2—H2	0.9300	C9—H9	0.9300
C3—N2	1.377 (2)	C10—C9^i^	1.378 (2)
C3—H3	0.9300	C10—H10	0.9300
C4—N3	1.324 (2)	C11—O2	1.368 (2)
C4—C5	1.377 (2)	C11—C13^ii^	1.383 (2)
C4—H4	0.9300	C11—C12	1.383 (3)
C5—C6	1.375 (2)	C12—C13	1.382 (2)
C5—N2	1.415 (2)	C12—H12	0.9300
C6—N4	1.329 (2)	C13—C11^ii^	1.383 (2)
C6—H6	0.9300	C13—H13	0.9300
C7—N3	1.321 (2)	O1—H1A	0.8200
C7—N4	1.329 (2)	O2—H2A	0.8200
			
N1—C1—N2	112.37 (16)	C10^i^—C9—C8	120.72 (16)
N1—C1—H1	123.8	C10^i^—C9—H9	119.6
N2—C1—H1	123.8	C8—C9—H9	119.6
C3—C2—N1	110.82 (15)	C9^i^—C10—C8	120.67 (16)
C3—C2—H2	124.6	C9^i^—C10—H10	119.7
N1—C2—H2	124.6	C8—C10—H10	119.7
C2—C3—N2	105.99 (16)	O2—C11—C13^ii^	117.69 (16)
C2—C3—H3	127.0	O2—C11—C12	122.90 (15)
N2—C3—H3	127.0	C13^ii^—C11—C12	119.40 (16)
N3—C4—C5	122.56 (17)	C13—C12—C11	120.52 (16)
N3—C4—H4	118.7	C13—C12—H12	119.7
C5—C4—H4	118.7	C11—C12—H12	119.7
C6—C5—C4	116.74 (16)	C12—C13—C11^ii^	120.08 (17)
C6—C5—N2	121.95 (15)	C12—C13—H13	120.0
C4—C5—N2	121.31 (15)	C11^ii^—C13—H13	120.0
N4—C6—C5	121.87 (17)	C1—N1—C2	104.87 (15)
N4—C6—H6	119.1	C1—N2—C3	105.95 (14)
C5—C6—H6	119.1	C1—N2—C5	126.48 (14)
N3—C7—N4	126.88 (17)	C3—N2—C5	127.57 (15)
N3—C7—H7	116.6	C7—N3—C4	115.79 (16)
N4—C7—H7	116.6	C7—N4—C6	116.16 (16)
O1—C8—C9	118.15 (15)	C8—O1—H1A	109.5
O1—C8—C10	123.22 (15)	C11—O2—H2A	109.5
C9—C8—C10	118.61 (16)		
			
N1—C2—C3—N2	0.1 (2)	C3—C2—N1—C1	−0.5 (2)
N3—C4—C5—C6	−1.2 (3)	N1—C1—N2—C3	−0.7 (2)
N3—C4—C5—N2	178.71 (17)	N1—C1—N2—C5	178.84 (15)
C4—C5—C6—N4	0.4 (3)	C2—C3—N2—C1	0.3 (2)
N2—C5—C6—N4	−179.50 (16)	C2—C3—N2—C5	−179.20 (16)
O1—C8—C9—C10^i^	179.04 (17)	C6—C5—N2—C1	154.15 (18)
C10—C8—C9—C10^i^	0.3 (3)	C4—C5—N2—C1	−25.8 (3)
O1—C8—C10—C9^i^	−178.97 (17)	C6—C5—N2—C3	−26.4 (3)
C9—C8—C10—C9^i^	−0.3 (3)	C4—C5—N2—C3	153.65 (18)
O2—C11—C12—C13	178.57 (16)	N4—C7—N3—C4	−0.3 (3)
C13^ii^—C11—C12—C13	−0.1 (3)	C5—C4—N3—C7	1.2 (3)
C11—C12—C13—C11^ii^	0.1 (3)	N3—C7—N4—C6	−0.4 (3)
N2—C1—N1—C2	0.7 (2)	C5—C6—N4—C7	0.3 (3)

Symmetry codes: (i) −*x*, −*y*+1, −*z*+1; (ii) −*x*, −*y*, −*z*.

### Hydrogen-bond geometry (Å, °)

**Table:**

*D*—H···*A*	*D*—H	H···*A*	*D*···*A*	*D*—H···*A*
O1—H1A···N1^iii^	0.82	1.96	2.764 (2)	168.
O2—H2A···N4^iv^	0.82	2.02	2.835 (2)	174.

Symmetry codes: (iii) *x*, *y*, *z*+1; (iv) −*x*+1, −*y*+1, −*z*+1.
